# Functional outcome in patients with hip fracture from 2008 to 2018, and the significance of hand-grip strength – a cross-sectional comparative study

**DOI:** 10.1186/s12877-023-04398-9

**Published:** 2023-10-23

**Authors:** Noelle Probert, Åsa G. Andersson

**Affiliations:** 1 School of Medical Sciences, Faculty of Medicine and Health, Örebro University, Örebro, SE70182 Sweden; 2 Department of Radiology, Centre for Clinical Research, Region Värmland, Karlstad, SE65182 Sweden; 3 Department of Geriatrics, School of Medical Sciences, Faculty of Medicine and Health, Örebro University, Örebro, Sweden

**Keywords:** Hip fracture, Comorbidity, Surgical method, Development, Functional outcome, Hand-grip strength

## Abstract

**Background:**

Incidence of hip fracture is estimated to rise, increasing demands on healthcare. Our objective was to compare patients with hip fracture, a decade apart, regarding surgical characteristics and functional outcome in relation to morbidity. A secondary aim was to analyse postoperative hand-grip strength (HGS) in relation to walking ability 4 months postoperatively.

**Methods:**

This is a cross-sectional comparative study of patients with hip fracture, included in 2008 (n = 78) and 2018 (n = 76) at Örebro University Hospital. Patient-data (age, gender, morbidity, fall-circumstances, fracture, surgical characteristics, and length of stay) were collected from medical records. HGS was measured postoperatively. Data on functional outcome in terms of housing, walking ability and need of walking aids at 4 months postoperatively was collected from the Swedish Hip Fracture Register RIKSHÖFT. Statistical analyses adapted were hypothesis tests and regression analysis.

**Results:**

Patients in 2018 presented a significantly higher morbidity than patients in 2008 and there were significant differences in adapted surgical methods. Functional outcome at 4-months postoperatively was analysed by logistic regression where Cohort 2018 was associated with higher odds of independent walking ability (OR 5.7; 95%CI 1.9–17.2) and not needing any walking aids (OR 5.1; 95%CI 1.9–17.2). Postoperative HGS was higher among patients in 2018 and a multiple regression analysis revealed a significant association between HGS and walking ability at 4 months postoperatively.

**Conclusions:**

This study supports the since previously reported development in hip fracture surgery in Sweden while also presenting that functional outcome seems to have improved despite a concomitant increase in morbidity. Results suggest an improvement in postoperative HGS, predicting walking ability at 4 months postoperatively.

## Introduction

Hip fracture is a devastating condition causing excess mortality in older people [1]. Sweden represents one of the highest incidences worldwide with approximately 16 000 registered hip fractures annually and a lifetime risk of approximately 20% in women and 10% in men [2–4]. Incidence is expected to rise with longevity, increasing the demands on healthcare in treatment and patient management [5].

Hip fractures are grossly classified as those of the femoral neck or trochanteric fractures and the major surgical methods used are arthroplasty or osteosynthesis [6]. Arthroplasty is associated with a longer surgical duration and length of stay (LOS) but potentially also a better functional outcome postoperatively [6, 7]. It is unclear whether the methods differ regarding postoperative mortality, but a higher failure rate has been seen for osteosynthesis, requiring further surgery [7]. Delayed surgery is associated with increased medical complications, mortality and functional outcome [8–11] and Swedish national guidelines recommend that 80% of patients are operated within 24 h after arrival at a healthcare facility [2].

During the last decades in Sweden there has been a development towards arthroplasty from osteosynthesis in treatment of femoral neck fractures and an increase of intramedullary nailing regarding methods of osteosynthesis [6, 9, 12]. At the same time, LOS has decreased and time to surgery has remained unchanged with approximately 60% of patients operated within 24 h [13]. However, despite this potential development, according to longitudinal studies, subsequent functional outcome at 4 months postoperatively seems to have remained unaltered [6, 13]. A suggested reason for this is a concomitant increase in individual comorbidity-burden and potential frailty within the population [6, 13, 14]. Internationally, a few studies do present an association between individual comorbidities and functional outcome in patients with hip fracture although differing in follow-up time and measurement of outcome [15–18]. Furthermore, increased age (> 85) is associated with worser functional outcome and increased frailty in previous studies and has also been presented as an independent risk factor of mortality post hip fracture despite level of frailty and comorbidity [19].

Early functional evaluation in hip fracture patients has an important prognostic value and hand-grip strength (HGS) is an objective and easily measured surrogate for whole body- and specifically lower-limb strength [20, 21] in addition to being an important factor in assessment of frailty [22] and sarcopenia [23]. The European Working Group on Sarcopenia in Older People (EWGSOP) revised the criteria for sarcopenia in 2019, providing validated cut-off values for hand-grip weakness in older people [23]. HGS has been positively associated with functional outcome in hip fracture patients by a few studies [24–26] although to our knowledge not in a Swedish population and none have evaluated a possible association with walking ability at 4 months postoperatively using the EWGSOP2-criteria [23].

This study sought to compare patients with hip fracture from 2008 to 2018 regarding surgical characteristics and 4-month postoperative functional outcome in relation to individual morbidity. A secondary aim was to compare postoperative HGS in relation to walking ability at 4 months postoperatively.

## Methods

### Study design and population

This was a prospective cross-sectional comparative study where all patients going through surgery due to acute hip fracture diagnosed with ICD-10 codes S72.0 (femoral neck fracture), S72.1 (pertrochanteric fracture) or S72.2 (subtrochanteric fracture) during the periods of Oct 2008 to Feb 2009 and Feb 2018 to Jun 2018 at Örebro University Hospital, were consecutively invited to participate. A written consent signed firstly by the patient or, if possible, secondarily by next of kin was acquired for all included participants. No exclusion criteria existed.

### Data collection, variables, and measurements

Individual patient data (age, gender, fall-circumstances, fracture-type, measures of morbidity, time to surgery, surgical-method, LOS, and mortality) were collected from individual medical records using a standardized review protocol.

Age was calculated from year of birth. Gender was male or female. Morbidity was assessed by: preoperative American Society of Anaesthesiologist Classification (ASA-class) [27], individual comorbidities (verified in the medical records according to ICD-10 codes where all Elixhauser comorbidities were evaluated [28]), and multimorbidity, defined as having ≥ 3 comorbidities. Time to surgery was defined as hours from radiology statement of hip fracture to time of surgery. Surgical methods were verified in the medical records according to the Swedish translation of the collective Nordic operational codes: NOMESCO classification of surgical procedures (NCSP69).

HGS was measured with a hand dynamometer (Jamar) in kilograms (kg). The best attempt of three after assessment of both hands was evaluated, cut-off < 27 kg for men and < 16 kg for women according to the EWGSOP2-criteria [23]. All measurements of HGS were carried out bedside before discharge within the first seven days postoperatively by a few licensed physiotherapists, trained in the method. Measurements were conducted in everyday clinical life and included patients received healthcare as well as in-hospital physiotherapy according to normal routines.

### Functional outcome

Functional outcome at 4 months postoperatively was assessed by three measurements: housing, walking ability and the need of walking aids. This data (both pre-fracture and at 4 months postoperatively) in addition to data on reoperation was extracted from the Swedish Hip Fracture Register RIKSHÖFT (SHR), a national, clinical, quality register with an estimated coverage of > 80% of all hip fractures in Sweden [2]. The different categories of housing, walking aids and walking ability registered were recoded to facilitate the analysis and to improve clinical applicability. “Ordinary housing” corresponded to patients living in their own home while “institutionalized housing” corresponded to any service-housing, rehabilitation-unit/convalescent home, acute hospital or other. “Independent walking ability” corresponded to being able to walk independently both indoors and outdoors while “dependent walking ability” corresponded to needing to be accompanied to walk outdoors and/or indoors. “No need of walking aids” corresponded to not needing any walking aids at all and “walking aids” corresponded to the need of any walking aids except for wheelchair which was considered and presented separately.

### Statistical analysis

Differences in age, surgical length and LOS were analysed by independent sample *t* test, differences in comorbidity-count were analysed by the Mann-Whitney *U* test and differences in categorical variables with the chi-square test.

Unadjusted and adjusted logistic regression were performed for the three different functional outcomes in terms of housing, walking aids and walking ability to compare the two cohorts. Adjustment was made for confounders as presented in Table 1. All variables were evaluated on categorical scale. Logistic regression gives odds ratio (OR) with 95% confidence intervals (CI) as association measures. A P-value lower than 0.05 was considered statistically significant and all analyses were performed in IBM SPSS (Armonk, NY, USA) version 25.

**Table 1:** Patient characteristics, surgical characteristics and postoperative HGS of Cohort 2008 and 2018

		Cohort 2008 *n* = 78	Cohort 2018 *n* = 76	P
Patient characteristics – pre-fracture				
Age, mean (SD), years		81(11)	80(12)	0.68
	Age ≥ 80, *n (%)*	50(64)	45(59)	0.53
Gender, female, *n* (%)		49(63)	45(59)	0.65
Comorbidity-count, median (IQR)		1(1)	2(1)	< 0.01
	Multimorbidity ^a^, *n* (%)	10(13)	21(31)	0.02
ASA-class, *n* (%)	1	10(13)	5(7)	
	2	37(47)	25(33)	< 0.01
	3	29(37)	34(45)	
	4	2(3)	12(16)	
Housing, *n* (%)	Ordinary	65(83)	70(92)	0.10
	Institutionalized	13(17)	6(8)	
Walking ability, *n* (%)	Independent	51(65)	53(70)	
	Dependent	22(28)	20(26)	0.74
	Could not walk	5(6)	3(4)	
Walking aids, *n* (%)	None	32(41)	38(51)	
	Walking aid	41(53)	35(47)	0.33
	Wheelchair	5(6)	2(3)	
Fracture and surgery				
Coplanar-fall-related fracture, *n* (%)		76(97)	71(93)	0.23
Type of fracture, *n* (%)	S72.0	41(53)	37(49)	
	S72.1	31(40)	31(41)	0.79
	S72.2	6(8)	8(11)	
Surgery within 24 h, *n* (%)		39(50)	32(42)	0.33
Surgical method, *n* (%)	Osteosynthesis with pins, nails, screws, and plates	60(77)	42(55)	
	Intramedullary nail	3(4)	13(17)	0.01
	Hemi-arthroplasty	13(17)	14(18)	
	Total arthroplasty	2(3)	6(8)	
	Flail joint	0(0)	2(3)	
Length of stay, mean (SD), days		10(5)	9(4)	0.70
Postoperative HGS		***n*** **= 69**	***n*** **= 57**	
HGS, mean (SD), kg		21(11)	26(11)	0.01
HGS under cut-off ^b^		33(48)	11(19)	< 0.01

^a^, ≥ 3 comorbidities; ^b^, < 27 kg for men and < 16 kg for women; Abbreviations: SD: standard deviation; IQR: Inter Quartile Range; ASA: American Society of Anaesthesiologists; HGS: hand-grip strength

## Results

### Participants

**Fig. 1:**
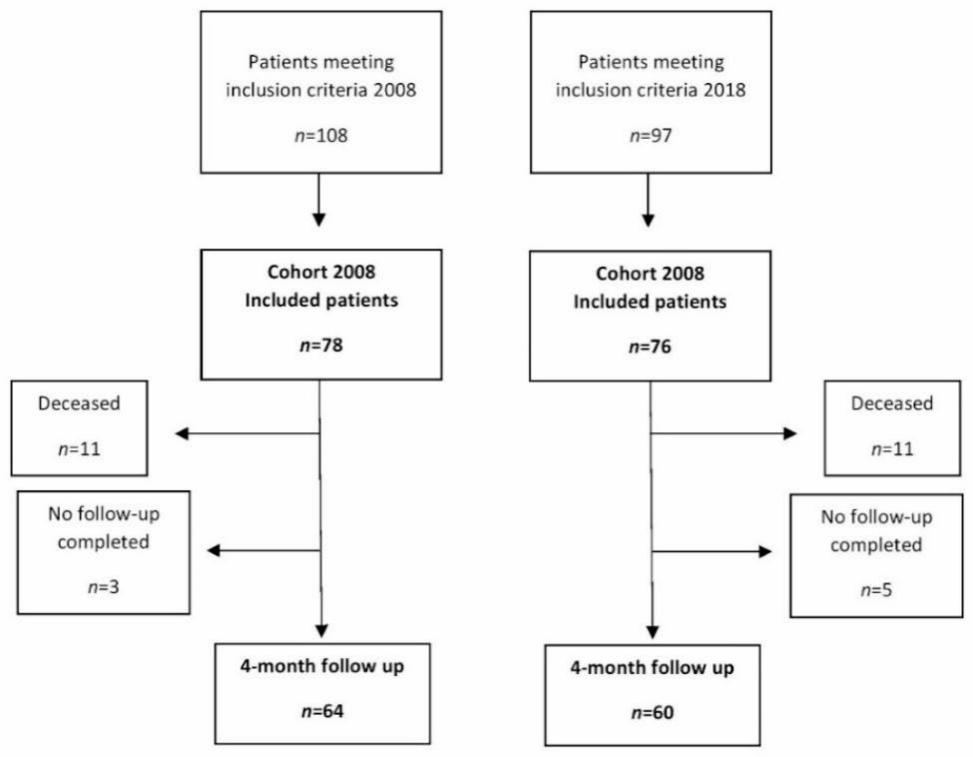
Patients included at baseline and in the follow-up at 4 months postoperatively

A total of 108 and 97 patients met the inclusion criteria in 2008 and 2018, respectively. In 2008, 30 patients did not give their consent for inclusion and in 2018 the corresponding number was 21, leaving 78 patients included in 2018 and 76 patients in 2018, see Fig. 1. Impaired ability to give consent due to cognitive state in the acute setting was the most common reason for non-inclusion in both cohorts. No cognitive screening tests were performed. There was no significant difference in gender, comorbidity or time to surgery when comparing the included cohorts with the non-included groups in 2008 and 2018. The mean age of the included cohort in 2008 was 81 years compared to 84 years in the non-included group, presenting no significant difference (P = 0.26). To the contrary, the non-included group in 2018 presented a significantly higher mean age of 87 compared to the mean age of 80 in the included cohort (P = 0.007).

### Baseline characteristics

As presented in Table 1, the cohorts where alike in terms of age and gender. There were no significant differences in pre-fracture housing, walking-aids, or walking ability.

**Fig. 2:**
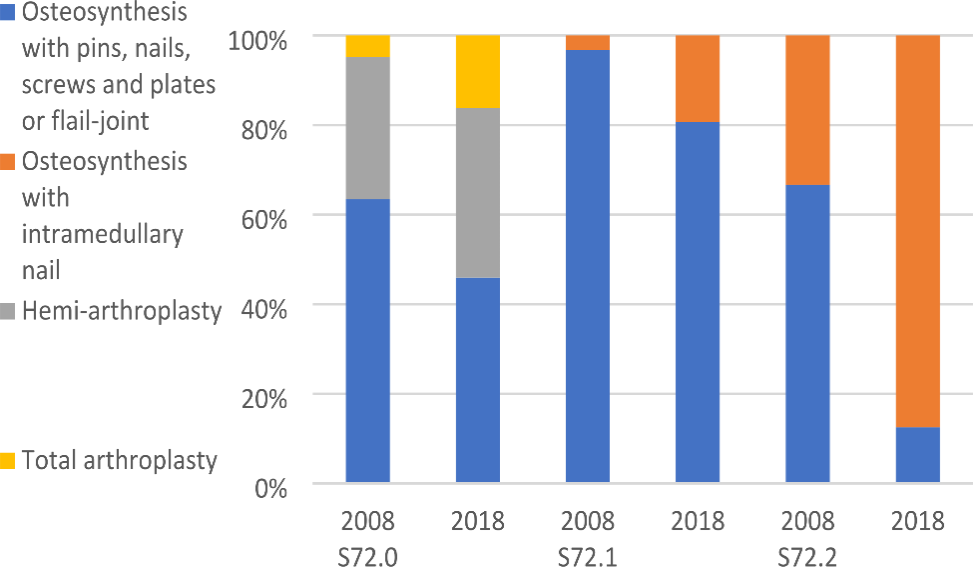
Surgical methods in relation to fracture-type in Cohort 2008 and Cohort 2018. S72.0, femoral neck fracture; S72.1, pertrochanteric fracture; S72.2, subtrochanteric fracture

The cohorts differed significantly in preoperative morbidity in terms of median comorbidity-count, multimorbidity and ASA-class of 3–4, where Cohort 2018 presented significantly higher values. No patients were assessed with a preoperative ASA-class higher than 4. In addition, there were significant differences regarding surgical method where arthroplasty and osteosynthesis with an intramedullary nail was more common in 2018 than 2008, also further presented according to fracture-type in Fig. 2. Surgery within 24 h and LOS remained unaltered.

Postoperative HGS was assessed in 69 patients in Cohort 2008 with a loss of nine (three due to patient-related conditions, one discontinued participation, one early death and four unspecified) and in 57 patients in Cohort 2018 with a loss of 19 (eight due to patient-related conditions, one declined participation, six occasions due to lack of resources and three unspecified). When the total fallout group of 28 patients was compared with the group of 126 patients where HGS was measured there were no significant differences in gender (P = 0.41), mean age (P = 0.19) or mean number of comorbidities (P = 0.35). In 2008 the average time between surgery and measurement of HGS was 6 days (SD 2) and in 2018 the average time was also 6 days (SD 4), (*p* = 0.15). The mean HGS was significantly higher in Cohort 2018 and there were significantly more patients with a HGS under cut-off in Cohort 2008, see Table 2.

**Table 2:** Functional outcome at the 4-month follow-up

		Cohort 2008	Cohort 2018	P
		***n*** **= 64**	***n*** **= 60**	
Housing, *n* (%)	Ordinary	50(78)	54(90)	0.07
	Institutionalized	14(22)	6(10)	
Walking ability, *n* (%)	Independent	36(56)	25(42)	
	Dependent	5(8)	2(3)	0.09
	No walking ability	23(34)	33(55)	
Walking aid, *n* (%)	None	9(14)	13(22)	
	Walking aid	49(77)	45(75)	0.25
	Wheelchair	6(9)	2(3)	

As presented in Fig. 1, 11(14%) patients in Cohort 2008 and 11(14%) patients in Cohort 2018 died before the follow-up at 4 months postoperatively, P = 0.95. In addition, for three (4%) patients in Cohort 2008 and five (7%) patients in 2018 no follow-up was completed, P = 0.45. The most common reason for no follow-up was that the patient could not be reached via telephone.

There were no significant differences in the three different measures of functional outcome of housing, walking ability and the use of walking aids between Cohort 2008 and Cohort 2018 at the 4-month follow-up, see Table 3. In addition, four (5%) patients in 2008 and one (1%) patient in 2018 were re-operated within follow-up (P = 0.18). All the re-operated patients in 2008 had been primarily treated with osteosynthesis with pins or screws due to femoral neck fractures where three patients (two fracture-dislocations and one pseudoarthrosis) were re-operated with a hemiarthroplasty and one patient received a total arthroplasty due to caput necrosis. The single re-operated patient in 2018 was primarily treated by osteosynthesis with a twin-hook due to a per-trochanteric fracture and was re-operated due to a peri-implant fracture with re-osteosynthesis.

**Table 3:** Unadjusted and adjusted logistic regression for the functional outcomes at the 4-month follow-up

		*n* (%)	Unadjusted *n* = 124		Adjusted *n* = 124	
			**OR (95%CI)**	**P**	**OR (95%CI)**	**P**
Ordinary housing at follow-up						
	Cohort 2018		2.5 (0.9–7.1)	0.08	2.1 (0.6–7.4) ^a^	0.30
	Cohort 2008		reference		reference	
Independent walking ability at follow-up						
	Cohort 2018		2.2 (1.1–4.5)	0.03	5.7 (1.9–17.2) ^b^	< 0.01
	Cohort 2008		reference		reference	
No need of walking aids at follow-up						
	Cohort 2018		1.7 (0.7–4.3)	0.30	5.1 (1.0-26.4) ^c^	0.05
	Cohort 2008		reference		reference	

^a^ Adjusted for housing before fracture, gender, age, multimorbidity (≥ 3 comorbidities), ASA-class ≥ 3 and surgical method (arthroplasty or osteosynthesis)

^b^ Adjusted for walking ability before fracture, gender, age, multimorbidity (≥ 3 comorbidities), ASA-class ≥ 3 and surgical method (arthroplasty or osteosynthesis)

^c^ Adjusted for walking aid before fracture, gender, age, multimorbidity (≥ 3 comorbidities), ASA-class ≥ 3 and surgical method (arthroplasty or osteosynthesis)

A multiple logistic regression analysis was performed for the three functional outcomes at 4 months postoperatively, see Table 3. The unadjusted analysis revealed a significant association between Cohort 2018 and independent walking ability, remaining significant in the adjusted analysis. The adjusted analysis also revealed a significant association between Cohort 2018 and the outcome of not needing any walking aids.

**Fig. 3:**
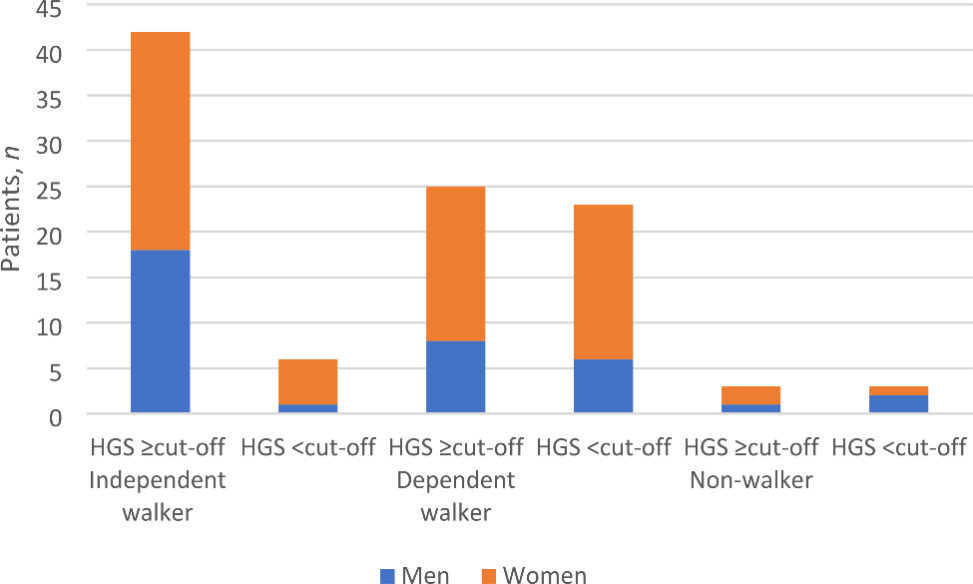
HGS measured postoperatively before discharge, presented in relation to reported walking ability 4 months postoperatively. Cut-off: < 27 kg for men and < 16 kg for women. Abbreviations: HSG, hand-grip strength

The comparison of postoperative HGS and functional outcome at follow-up included 102 patients (58 patients in 2008 and 44 patients in 2018) due to reasons as described earlier. When comparing postoperative HGS according to the cut-off values of EWGSOP2 with walking ability at the 4-month follow-up there were more independent walkers among the patients who had a HGS over cut-off in both cohorts, further described in Fig. 3. A potential association between postoperative HGS and an independent walking ability at the 4-month follow-up was analysed in a logistic regression analysis adjusted for age and gender revealing a significant OR of 5.8 (CI1.7-17.4, P = < 0.01), see Table 4.

**Table 4:** Unadjusted and adjusted logistic regression for independent walking ability, presented in relation to postoperative HGS

		*n* (%)	Unadjusted *n* = 102		Adjusted *n* = 102	
			**OR (95%CI)**	**P**	**OR (95%CI)**	**P**
Independent walking ability at follow-up						
	HGS over cut-off ^a^		6.5 (2.4–17.8)	< 0.01	5.8 (1.7–17.4) ^b^	< 0.01
	HGS under cut-off		reference		reference	

^a^ Cut-off < 27 kg for men and < 16 kg for women; ^b^ Adjusted for age and gender. Abbreviations: HGS: hand-grip strength; OR: odds ratio; CI: confidence interval

## Discussion

Results of this cross-sectional comparative study present that the 4-month postoperative functional outcome in hip fracture patients potentially has improved during the last decade in Sweden despite a concomitant increase in morbidity and that postoperative HGS is associated with walking ability at 4 months postoperatively. The study is limited by its small sample-size and observational design although still contributing to knowledge-gaps of the Swedish hip fracture population and further highlighting the potential prognostic value of postoperative HGS.

A majority of the patients in this study were women with a mean age of 80–81 years which is in line with other studies and national data [6, 12]. Patients in 2018 presented a higher morbidity-level in terms of an increased comorbidity-burden, multimorbidity and preoperative ASA-class compared to patients in 2008 which has also been reported by previous studies both nationally and internationally [13, 29]. Our results are also in line with previous studies in reporting a shift in choice of surgical methods during the last decades in Sweden as well as in other countries. The shift being an increased use of intramedullary nailing in trochanteric fractures and arthroplasty in femoral neck fractures [6, 12]. In addition, our study presented no statistically significant difference regarding surgeries performed within 24 h, a trend also supported by larger Swedish longitudinal studies [13]. This is potentially related to the concomitant increase in morbidity where preoperatively unstable medical conditions is a known contributor to prolonged time to surgery [30]. The cohorts did not differ significantly regarding LOS, although our results do indicate a decrease which is also what other studies have reported over time in Sweden [13]. The 4-month mortality-rate was 14% in 2008 respectively 14% in 2018 (P = 0.95) which is similar to but slightly higher than what has been reported in other Swedish studies [13, 31]. An age over 85 years has been presented as an independent risk factor for 1-year mortality in patients with hip fracture by previous studies [19] although, in line with this, age did not differ significantly between the cohorts in this study.

There were no significant differences between Cohort 2008 and Cohort 2018 regarding the three measures of functional outcome, see Table 3, also supported by national data [6, 13]. However interestingly, the results of the logistic regression analysis for the three functional outcome measures in this study (see Table 3) revealed that after adjustment for preoperative functional status, age, gender, surgical method, and morbidity in terms of ASA-class and multimorbidity, the odds of being an independent walker and not needing any walking aids at the 4-month follow-up were 5.7 (95%CI 1.9–17.2) respectively 5.1 (95%CI 1.0-26.4) times significantly higher in 2018 than in 2008. The unadjusted analysis also presented a significant association between independent walking ability and patients in Cohort 2018 with an unadjusted OR of 2.2 (95%CI 1.1–4.5), although the level of significance and the odds ratio increased after adjustment. These results do suggest that the increased morbidity in Cohort 2018 seems to be affecting the patients’ recovery negatively and, in relation to earlier studies, potentially highlighting that the development over time regarding surgery and management of patients with hip fracture in Sweden has in some aspects been successful despite not being directly apparent in figures of functional outcome in previous studies, lacking data on individual comorbidity-burden [6, 13]. However, evidently this study has not considered all potential confounders.

Patients in Cohort 2018 had a significantly higher postoperative mean HGS and hand-grip weakness was significantly lower than in Cohort 2008 according to the cut-off values of EWGSOP2 [23]. These particular findings are discussed in relation to the increased comorbidity-burden and unaltered mortality in another study based on the same population as this study, published in 2020 [14]. A multiple logistic regression analysis revealed that patients who had a HGS over cut-off at discharge had significantly 5.8 (CI 1.7–17.4) times higher odds of being independent walkers at the 4-month follow-up after adjustment for age and gender, see Table 4. Previous studies support these results although differing in their follow-up time and measurement of functional outcome [24, 25, 32, 33]. Savino et al. found that a higher preoperative HGS was significantly correlated with a higher probability of independent walking recovery withing the first year postoperatively [32]. Milman et al. found that HGS as a continuous variable, as well as dichotomized according to the cut-off values by EWGSOP2, significantly predicted the success of rehabilitation in patients with hip fracture [33]. Di Monaco et al. and Selakovic et al. found significant correlations between postoperative HGS and better performance in activities of daily living up to six months postoperatively [24, 25] where Selakovic et al. also defined hand-grip weakness according to the definition by EWGSOP2 [24]. Considering this, our results contribute to and further underline the prognostic value of HGS, a quick and easily measured surrogate for whole body strength, not limited to patients with walking ability in the immediate postoperative phase [20, 21]. Furthermore, these findings also highlight the importance of physical activity and interventions to maintain muscle strength in the older population, considering the effect on postoperative functional outcome.

### Limitations and strengths

Results of this study are limited by the small sample-size and observational design. Most data were collected from medical records where the risk of error in documentation cannot be disregarded. Data on functional outcome at follow-up was collected from the SHR where follow-up data was initially collected via phone-conversations with patients or close relatives by use of a questionnaire and the risk of outcome misclassification cannot be completely ruled out. Furthermore, the non-included patients and fallout of data of this study is a limiting factor. The non-included group in 2018 was significantly older than the included cohort (p = 0.007) and inclusion could possibly have affected results. In addition, a total of 28 patients were not included in the measurement of postoperative HGS while three patients in 2008 and five patients in 2018 were not included in the follow-up which could also have affected results on HGS and functional outcome. The follow-up time of 4 months was adapted since it is the official follow-up time used by the SHR, although, also supported by previous studies as a valid time for assessing functional outcome in patients with hip fracture [34]. Furthermore, this study lacks data on individually performed in-hospital and post-discharge physiotherapy which of course could have interfered with results. A strength of this study is that it had no exclusion criteria in turn contributing to correctly portraying clinical reality. In addition, this study assessed patients through both registered data, individual data from medical records and bedside anthropometric measurements such as HGS, not possible in larger register-based studies. To our knowledge, this is the first cross-sectional study in Sweden assessing functional outcome after hip fracture surgery in relation to individual comorbidity-burden as well as assessing the potential predictive value of HGS in functional outcome.

## Conclusion

In conclusion, by comparing patients with hip fracture, a decade apart, this study supports the since previously reported developments in hip fracture-surgery and hospitalization in Sweden while also presenting that functional outcome seems to have improved despite a concomitant increase in morbidity. Results suggest an improvement in postoperative HGS, significantly associated with walking ability at 4 months postoperatively.

## Data Availability

The datasets used and/or analyzed during the current study are available from the corresponding author on reasonable request.

## Abbreviations

LOS: Length of stay
HGS: Hand-grip strength
EWGSOP: European Working Group on Sarcopenia in Older People
ASA–class: American Society of Anaesthesiologist Classification
SHR: Swedish Hip fracture Register
